# *SoEasy*: A Software Framework for Easy Hardware Control Programming for Diverse IoT Platforms

**DOI:** 10.3390/s18072162

**Published:** 2018-07-05

**Authors:** Junyoung Lee, Gwang-il Park, Jong-ha Shin, Jin-hae Lee, Cormac J. Sreenan, Seong-eun Yoo

**Affiliations:** 1 Department of Computer and Communication Engineering, Daegu University, Gyeongsan 38453, Korea; ljy908@daegu.ac.kr; 2 School of Computer and Communication Engineering, Daegu University, Gyeongsan 38453, Korea; pki054@daegu.ac.kr (G.-i.P.); prattlesjh@daegu.ac.kr (J.-h.S.); centipede486@daegu.ac.kr (J.-h.L.); 3 Department of Computer Science, University College Cork, Cork T12 YN60, Ireland; cjs@cs.ucc.ie

**Keywords:** visual programming tool, Internet of Things, Web of Things, IoT development tool

## Abstract

Many Internet of Things (IoT) applications are emerging and evolving rapidly thanks to widespread open-source hardware platforms. Most of the high-end open-source IoT platforms include built-in peripherals, such as the universal asynchronous receiver and transmitter (UART), pulse width modulation (PWM), general purpose input output (GPIO) ports and timers, and have enough computation power to run embedded operating systems such as Linux. However, each IoT platform has its own way of configuring peripherals, and it is difficult for programmers or users to configure the same peripheral on a different platform. Although diverse open-source IoT platforms are widespread, the difficulty in programming those platforms hinders the growth of IoT applications. Therefore, we propose an easy and convenient way to program and configure the operation of each peripheral using a user-friendly Web-based software framework. Through the implementation of the software framework and the real mobile robot application development along with it, we show the feasibility of the proposed software framework, named *SoEasy*.

## 1. Introduction

We have witnessed recently how open-source hardware platforms are widely used in the design of embedded systems like the Internet of Things (IoT). There are various kinds of open-source hardware platforms, from low-cost eight-bit microcontroller-based ones to high-performance ones with a 32-bit microcontroller. These hardware platforms were expensive in the past, but recently, with the help of advancements in semiconductor manufacturing technology, they have been getting more affordable and are obtained easily, thanks to the open-source distribution policy [1]. Therefore, everybody, beginners or experts in embedded systems, can easily design and implement their own IoT applications. However, when they start to develop new IoT applications, there is much information to know and understand; they need to know the detailed architecture of the hardware platforms and details about the peripherals used (e.g., UART, PWM, GPIO, etc.), as well as which interface to use with which sensors and actuators. However, it is not straightforward for a non-specialist to configure peripherals, each of which is designed and configured in a different way depending on the hardware platform.

For beginners who are unfamiliar with embedded software programming, some of the manageable embedded hardware platforms (Arduino [2], Raspberry Pi [3,4], Galileo [5,6], etc.) were introduced with a bunch of application programming interfaces (APIs) and libraries to control low-level hardware. However, users need to know some level of source coding. Furthermore, graphical visual programming tools [7] have been developed using graphical blocks or flowcharts, with which users simply develop embedded software. However, there are still limitations in developing some complex applications, such as extensibility and portability. Therefore, we propose the *SoEasy* software framework, which supports software development without complex source coding while helping users to develop a sophisticated application or extend or port an existing application to a different hardware platform. When using *SoEasy* to control peripheral devices (interfacing with sensors and actuators), developers do not need to know compiling and source-coding processes. To evaluate the software framework, we implemented the *SoEasy* framework, which is designed to be convenient, extensible and portable, and we demonstrated the ease and convenience of peripheral device control programming with *SoEasy*.

The following summarizes the contributions of this paper:

- Deriving the requirements (i.e., easiness, extensibility, and portability) for an easy programming framework, and the new software framework named *SoEasy*, which appears simple and easy to use for a beginner and provides sophisticated programming functions and services for an advanced user;
- Presenting the design for and results from the implementation of *SoEasy*, which addresses the aforementioned requirements and is verified in two representative open hardware platforms, ARM-based Raspberry Pi2 and X86-based Galileo;
- Evaluating the framework with a real application development procedure.

This paper is organized as follows. Section 2 introduces related work and derives the requirements for an easy, extensible and portable software development environment. Section 3 proposes the software framework based on the requirements, and Section 4 describes its implementation results. Section 5 evaluates *SoEasy*, and finally, the paper concludes with a summary and future work in Section 6.

## 2. Related Work

The IoT can be defined as the technologies that enable things or objects surrounding us to communicate and interact with one another for any meaningful action [8]. One of the application areas for the IoT is the smart home, and according to the technical advances, the Internet is connected to many household appliances around us. As a result, users can easily monitor and control Internet-connected home appliances with their smartphones. Recently, users have created many IoT systems by themselves, as there are many inexpensive embedded hardware platforms available. However, some users are not familiar with the development of IoT systems, so to support them, various approaches have been developed. One approach is based on the exposed API to access the provided libraries, which is quite conventional. Some of the examples of this approach are the MRAA [9] and hwio [10] libraries, which provide a variety of functions for the user’s convenience. However, they still require users to learn difficult programming languages. Another approach provides a user with a bunch of source code based on the demands of the user, but this approach has difficulties similar to the first approach. Another approach is based on visual programming tools, which make the programming easier than the previous approaches by offering some graphical tools, and users can develop programs by combining graphic elements. Our proposal is conceptually similar to visual programming, and we overview the last two approaches rather than the traditional method.

### 2.1. On-Demand Source Code Provision through the Web

The site called Temboo [11,12] provides features where a user downloads source code for IoT systems through the Web. From this site, a user can select the features required, and Temboo provides information on the necessary peripheral devices, schematics and, ultimately, the source code for IoT applications. Therefore, the user can develop IoT applications using them. However, when users want to modify or extend a feature from an existing application, they need to understand the relevant source code. Of course, to port an application to a new hardware platform, developers need source-level modification. Non-professional users find it difficult to analyze and modify source code. To provide them with *ease* and *extensibility*, *SoEasy* does not require users to analyze the source code when modifying an existing application, and users can modify the existing application to control the connected peripheral devices through the built-in Web User Interface (Section 4.2). In addition, *SoEasy* also provides portability (see Section 4.4.2) by exporting and importing the application, as well as the peripheral configurations. Since *SoEasy* does not require in-depth understanding of source code, a complete novice at programming can easily make an application to control external peripherals on IoT platforms, relieved of the need to configure peripheral ports and create source code.

### 2.2. Visual Programming Tools

There are visual programming tools using blocks for non-professionals. Among them, Mindstorms [13] was developed for educational purposes by the Massachusetts Institute of Technology (MIT) and Lego Systems, Inc. Users can make the various robots work through programming Mindstorms. However, the Mindstorms development tool has a disadvantage in that users cannot use platforms other than Lego Mindstorms. Blockly, another open source visual programming tool, is a JavaScript library for a visual programming environment developed by Google [14,15,16]. Blockly provides the features required to develop a Web application and an Android application through blocks. Based on the interlocking block coding concept, it generates the source code with options for JavaScript, Python, PHP, Lua or Dart. Since it is an extensible visual programming environment, and allows adding customer blocks, it can be used to develop an application for embedded systems [14]. Scratch was developed at MIT as a language for learning programming concepts. The basic concept is to create an animation by controlling a picture with a script by dragging and dropping blocks without writing source code [17]. Various blocks in Scratch are ready for variables, conditionals, iterations, and so on. The applications for Scratch are mainly animations and games. Developers use Scratch because it is easy to learn the concepts of programming, but it has limitations when developing realistic IoT applications. In contrast, *SoEasy* follows the visual programming concept of the aforementioned tools for ease of use, but *SoEasy* is a tool that can help users create more complex and realistic IoT applications.

Prior research using this visual programming trend was still dependent on the program development process. This method requires a process of combining the graphic elements implemented in block form and then converting and compiling the source code to generate the application program. In *SoEasy*, even though the visual programming method was used to make an application, it does not always require converting and compiling source code into a new executable. This study proposes a visual programming method that can add and configure external hardware devices and change the hardware configuration data in real time without converting and compiling any source code.

There are several Web-based or mobile application-based IoT development tools. Webduino [18,19] is a Web-based development tool using Blockly and JavaScript for the Arduino platform, and WebIOPi [20,21] is an IoT framework for Raspberry Pi to control and use input and output pins from a Web browser. Blynk [22,23] is a framework with iOS and Android mobile applications to control Arduino and Raspberry Pi over the Internet, and the Blynk mobile application needs to connect to a target IoT platform via the Blynk Cloud or a local Blynk server. It provides various widgets to access pins, and the widgets can be used to configure a graphical interface by dragging and dropping widgets. Webduino and WebIOPi support limited IoT platforms. WebIOPi and Blynk focus on simple input/output (I/O) pin access, rather than programming operation logic in an IoT platform. Like these IoT development tools, *SoEasy* provides an easy-to-use development environment. Added to this is *SoEasy*’s support for ARM- and X86-based multi-platforms, *extensibility* for sophisticated application development and portability to migrate an existing application to other platforms.

## 3. *SoEasy* Software Framework Design

**Figure 1:**
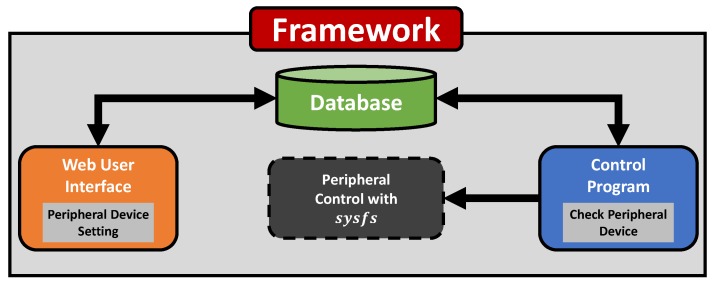
*SoEasy* framework.

From analysis of the existing work, we derived the requirements of a software framework for easy peripheral control programming: ease, extensibility and portability. To provide users with ease and convenience, we adopted Web technology, one of the most popular user interfaces, for *SoEasy*. Since most users are familiar with a Web interface, they can easily and conveniently make an application through the Web interface in *SoEasy* without doing any programming. In addition, Internet-enabled devices that have a Web browser can be a development host or a monitoring device. *SoEasy* also supports advanced services of extensibility (see Section 4.4.1) and portability (see Section 4.4.2), which facilitates an application software porting from one IoT platform to another. The *SoEasy* framework consists of a Database, Web User Interface and Control Program, as seen in Figure 1.

1. Web User Interface: This is the part that interfaces with users and updates the Database via user requests. The Web User Interface, with the help of a Web server, provides users with an intuitive Graphical User Interface (GUI) and helps non-expert users configure peripherals and easily create an application.
2. Database: The updated data affect the Control Program. The Database stores and manages all the information for *SoEasy*, including the configuration values for each built-in peripheral device, such as the general purpose input output (GPIO) controller and add-on function implementations.
3. Control Program: The Control Program is a service agent that controls the hardware platform according to the information in the Database.

Through these three components, the *SoEasy* framework helps users develop applications more conveniently and easily.

## 4. Implementation

**Figure 2:**
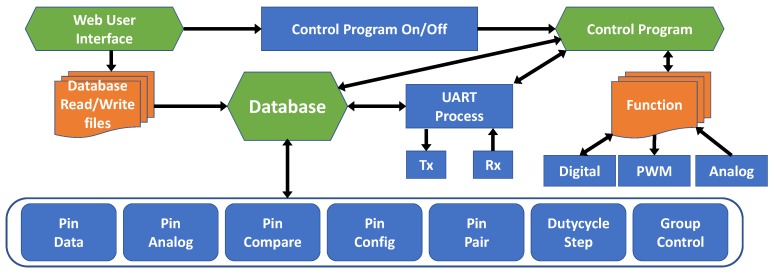
*SoEasy* detailed architecture.

To evaluate the *SoEasy* framework, we implemented it, as seen in Figure 2, in Linux for two representative open-source hardware platforms (Galileo Gen1/2 and Raspberry Pi). These two platforms are very popular and common Intel X86 architecture-based and ARM architecture-based platforms, respectively, and we implement *SoEasy* on these platforms. Figure 2 is the detailed architecture based on the *SoEasy* framework. In this section, we describe in detail the *SoEasy* functions and services that control peripheral devices.

### 4.1. Control Program

**Figure 3:**
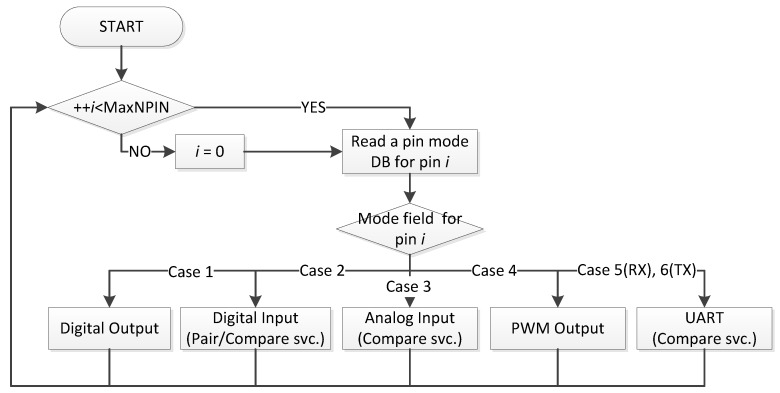
Overview of the Control Program.

The Control Program (Figure 3) performs the functions that are selected through the Web User Interface (Section 4.2). Based on the information stored in the database(Section 4.3), it configures the built-in peripherals and control registers to provide the user with specified functions, such as pulse width modulation (PWM), digital I/O, analog input and a universal asynchronous receiver and transmitter (UART) according to the user configuration. To do that, the Control Program accesses *sysfs* [24] in each case in Figure 3, a virtual file system provided by the Linux kernel, and modifies the information in the files in order to reflect the user requests. The Control Program spawns an independent child process for the UART interface to prevent any loss of UART data during execution of the main Control Program process. With these basic primitive functions, *SoEasy* provides more sophisticated services such as Pin-pairand Pin-compare.

#### 4.1.1. *SoEasy* Functions

The Control Program modifies the exported files in sysfs using low-level file input and output as follows. For the GPIO configuration, the Control Program exports the selected GPIO pins in order to create a symbolic link for the GPIO pins in the */sys/class/gpio* GPIO path in the *sysfs* directory. By writing “in” or “out” in the direction file for the pin in *sysfs*, according to the direction specified in the Database, the pin is configured to input or output mode, respectively. To read from or write to the pin, the Control Program reads the “value” file and stores it in the Database or it writes the value file to *sysfs* with the value from the Database. For analog input, the Control Program reads the analog value from the */sys/bus/iio/devices/iio:device0/in_voltage[Analog PIN] _value* analog path in sysfs. For PWM [25], the Control Program opens period and duty_cycle files in the */sys/class/pwm/pwmchip0/pwm[PIN number]* PWM path in sysfs and sets the period and the duty cycle according to the specified values.

For the UART, the Control Program uses the APIs in the WiringPIlibrary [26] to operate UART functionality and can communicate via UART by reading from and writing to the */dev/ttyS0* and */dev/ttyAMA0* UART paths. If the UART operates in the same process with the Control Program, UART data are prone to being lost. Therefore, *SoEasy* implements the UART and operates it separate from the main process.

#### 4.1.2. *SoEasy* Services

The most primitive service is Pin-pair service, which connects the selected source pin to a target pin. It helps any control program that controls a target pin by using selected source pin data. In Case 2 in Figure 3, the Control Program checks whether any input pin is set for this service, and it controls the target pin accordingly. The Pin-compare service is the implementation of an *if-then* statement in a programming language. It takes the input data from the pin specified by the pin number and checks it against a comparison value using a comparison operator. If the comparison condition is true, the pin data value is loaded to the target pin. The Control Program checks each input pin to see whether it is set to be a source of this service in Cases 2, 3 and 5 in Figure 3. If this service is set for the input pin, and the comparison condition is met, Control Program outputs the pin data to the associated target pin. For example, when a user creates an IoT application that monitors an environmental sensor, the Pin-compare service can be used to read the value of the current sensor and control a light-emitting diode (LED) by comparing the read value with the condition value. The Group-control service makes it easy to simultaneously control any pins in a group. *Tik-Tok* is another interesting service to control a pin using timers supported by the operating system. Users can create any applications requiring timer-relevant functions. *SoEasy* provides a timer page in the Web User Interface (Section 4.2), where users freely make a timer. To add a timer, users need to set the timer elements. When a timer function operates, the Timer_count_index is decreased each second. Therefore, when Timer_count_index is zero, Tik-Tok controls applications. An example of utilizing the Tik-Tok service is an application that blinks an LED every second. Users can make a variety of applications using the Tik-Tok service.

### 4.2. Web User Interface

**Figure 4:**
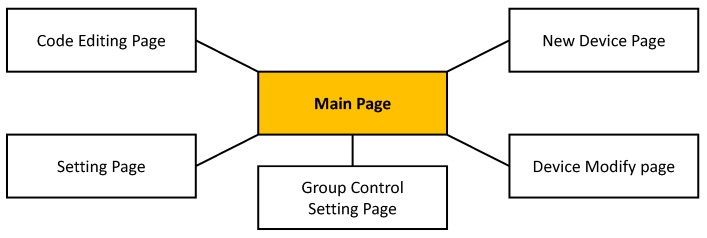
Web user interface architecture.

The Web User Interface provides pages where the user controls peripheral devices. According to Figure 4, the Web User Interface consists of six pages. In the Main Page, users can directly monitor and control the values of the peripheral devices. In the New Device Page, users choose a pin and its mode (e.g., digital, analog, PWM or UART). The Device Modify Page modifies the pin information the user has chosen. Users can modify the mode of the pin and add services, such as Pin-pair and Pin-compare services. In the Group-control Setting Page, users can add a Group-control service through the Web User Interface. Compared with other services, the Group-control service does not rely on the logic of the Control Program so much, but instead, for Group-control, the Control Program changes the output values for group pins by referring to the Group-control database (DB) table. Group-control can simultaneously be performed on a number of pins that users have set. *SoEasy* provides the Setting Page for changing the user interface’s background color and page title, and on the page, users can control the operating status (i.e., start or stop) of the Control Program. Finally, the Code Editing Page provides code editing of advanced services. When a user wants to modify some code in *SoEasy*, the user saves and compiles the modified code and can operate that compiled Control Program in the Setting Page.

### 4.3. Database

The Database plays one of the most central roles in *SoEasy*. Since *SoEasy* does not require any complex processing of application- specific data, this framework adopts a flat file structure-based Database, which makes portability support easier. The data of the Database are exchanged via the Web User Interface and Control Program. The Web User Interface was developed in PHP and runs under the privileges of a user named *www-data* in Linux without permission to access GPIO kernel device drivers. Therefore, *SoEasy* needs the Database to exchange the information between the Web User Interface and Control Program, which has higher privileges to access device drivers. Table 1 shows the fields in 10 database tables. When a user sets up a pin through the Web User Interface, the configuration data are stored in each field of the database table. The data are used by *SoEasy*’s Services and Functions. The Services provide various operations such as Pin-compare and Pin-pair. We describe some important DB tables in Table 1.

### 4.4. Advanced Functions

When professional users want to develop a more sophisticated application through *SoEasy*, they can feel the lack of functions and services provided. Therefore, *SoEasy* provides additional functions, such as code editing, export and import.

#### 4.4.1. Code Editing for Extensibility

*SoEasy* offers an advanced Web-based code editing function for expert users to modify existing functions or services, or to add a new function or service. This method has the advantage of being accessible from anywhere through any computing device with a Web browser, because it is based on Web technology. This is possible by allowing the user to edit the source code through the UI and to run the *make* utility (a common building tool) to compile any source code with the help of PHP scripts.

**Figure 5:**
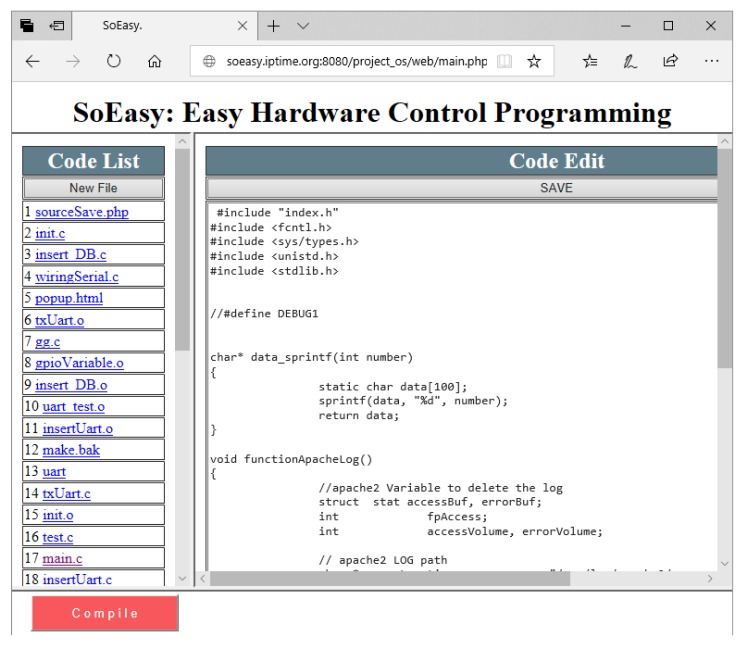
Code Editing function in a Web browser.

In addition, experienced developers can create a new module (e.g., a C source code for a compiled executable, a PHP script or an HTML document for a Web page) in this Web User Interface, compile a C source code module and run the new executable from the new module. Furthermore, developers can edit a new page module of the Web User Interface. The Database stores the data and the functions that can read or write a PIN value for a module program. The new module program can use Pin_data, Database and GPIO drivers to communicate with the Control Program. By sharing the data, a user can implement more complex applications. If you create a program that estimates ultrasonic sensor data, the Control Program and the sonar program operate at the same time. In some cases, a user wants to use a specialized peripheral device for an open-source hardware platform. For example, there is a Raspberry Pi camera [27] as a peripheral device. To use this device, the user creates a C source code module for an executable that controls the device and a Web page to show the contents (or video) captured by the Pi camera. In Figure 5, the Code Editing page in a Web browser, users can add a C source code module by clicking the ‘New File’ button, then writing their own source code in the right-hand editing box. After completing the source code, the user can compile the added module for the platform running *SoEasy* by clicking the ‘Compile’ button. When the user runs the executable file on the page, the user can operate the camera. This “Code Editing” function provides *SoEasy* with unlimited extensibility. It helps a user develop more applications conveniently.

#### 4.4.2. Export and Import for Portability

**Figure 6:**
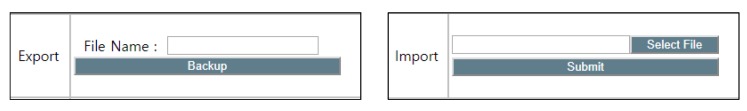
Portability. Left: specifying the exported hardware configuration; right: choosing the hardware configuration to import.

To move a developed application from one platform to a new platform, users are expected to set pin configurations and other additional configurations for the new platform. These procedures can be too cumbersome for some users. *SoEasy* provides an export and import function for hardware configuration information and services, such as Pin_pair, Pin_name, Pin_config and Pin_compare data in the DB. Therefore, users can move the entire application including pin configuration and services through a simple export and import process using the Web User Interface. For example, when a user wants to port a mobile robot application on a Galileo Gen 2 platform to a Raspberry Pi platform, the user specifies the configuration file name to export the hardware configuration and then clicks the Backup button in the left-hand figure in Figure 6; then, the export script creates the exported hardware configuration file. Now, the user can import the exported configuration file into the Raspberry Pi platform with the menu in the right-hand figure of Figure 6 by selecting the configuration file and clicking the Submit button. The last step is to remap the pins used on the previous platform for the new platform. This portability function provides a process that automatically matches the pins of the two different platforms. In this way, *SoEasy* supports portability between different hardware platforms through this function.

## 5. Evaluation

We evaluated the *SoEasy* framework from the following aspects:

- By comparison with the conventional approach and the existing Web or mobile application -based frameworks
- From a case study on designing a mobile robot control application in *SoEasy* to show the feasibility of the *SoEasy* framework.
- On the portability supported by *SoEasy*

### 5.1. Comparison with the Conventional Approach and the Existing Web- or Mobile Application-Based Frameworks

**Figure 7:**
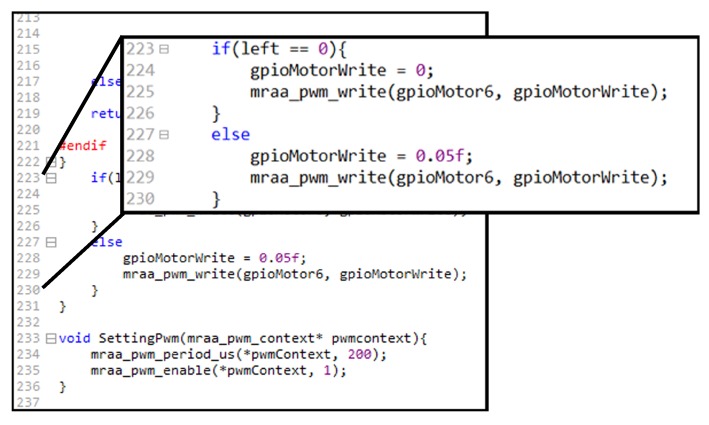
Conventional programming source code.

**Figure 8:**
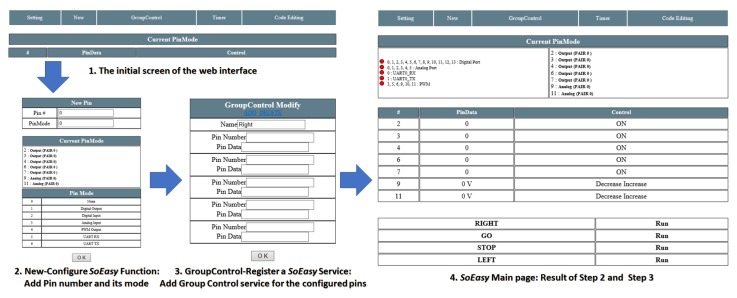
*SoEasy* programming interface.

Figure 7 shows a segment of Galileo Gen 1 application source code with a total of 239 lines for a project to control the mobile robot described in Section 5.2, while Figure 8 shows the workflow to make the same application in the Web User Interface for *SoEasy*.

Figure 7 and Figure 8, respectively, compare two different programming approaches using the conventional way (based on the MRAA library) and using *SoEasy* to build the same mobile robot control program, but these approaches are quite different in the following aspects. First, the conventional programming approach requires long-lined text coding. Even though developers can use libraries, such as hwio and MRAA, they need to understand the text-based programming language to perform complex tasks, such as mobile robot control and IoT applications. Unlike the conventional approach, if users adopt *SoEasy*, they can add pins for peripheral devices and configure the *SoEasy* Functions (Section 4.1.1) and the *SoEasy* Services (Section 4.1.2) using the Web User Interface, and the *SoEasy* framework runs the application accordingly. Like visual programming, this work process is much simpler than the conventional programming approach, while it provides the sophisticated control of the conventional approach.

We also compared the *SoEasy* framework to the existing Web-based and mobile application-based IoT development tools surveyed in Section 2. Table 2 summarizes the comparison results and shows that the *SoEasy* framework meets the design requirements (i.e., ease, extensibility and portability). We evaluated the ease of use by checking whether each framework requires users to have knowledge of any programming language syntax in order to create an application or not. For extensibility, we evaluated each framework from the following aspect: whether a framework itself can be extended or not. If a framework supports the ‘export and import’ (Section 4.4.2) of an application to a new platform, that framework has portability. Webduino has ease of use with the help of Blockly and offers portability, but it lacks multi-platform support and extensibility. WebIOPi is designed to monitor and control GPIOs of the Raspberry Pi platform through a Web browser. It is easy to use this framework to check and set the status of each GPIO, but the UI lacks programming support. Temboo is a commercial code-generating IoT development tool, and users can generate source code through a Web interface. Blynk is a mobile application-based framework requiring Blynk Cloud, and the extensibility of the tool is limited. *SoEasy* is a simple IoT framework and is easy to use, but it can be extended by providing the Code Editing function for an experienced developer, and it supports portability, which is evaluated in Section 5.3.

### 5.2. Mobile Robot Control Application

**Figure 9:**
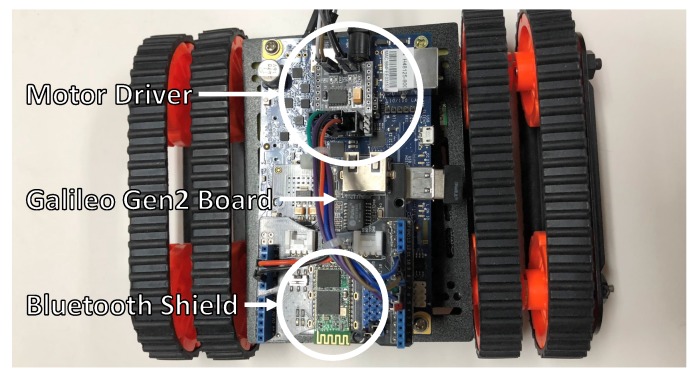
Mobile robot platform.

In order to evaluate *SoEasy*, we present the design workflow for an application that controls a mobile robot platform. Figure 9 is a mobile robot platform with an off-the-shelf chassis that can be controlled by *SoEasy*. It consists of a Galileo Gen 1 board [5], the mobile robot chassis (DG012-RP) [28], a motor driver (JMOD-Motor-1 [29]) and a Bluetooth shield [30]. First, we set up the hardware connections. The Bluetooth shield is connected to the Galileo Gen 1 board by the UART interface, and the motor driver, which uses digital I/O and PWM, is inserted between the Galileo board and the motors in the mobile robot chassis. Motor 1 controls the right wheels, while Motor 2 is responsible for the left wheels. We summarize the detailed pin connections in Table 3. Reception (Rx) and transmission (Tx) pins of the Bluetooth module are connected to Pin 0 and 1, respectively, and the control signals of the motor driver are connected to Pins 2, 3, 4, 6, 7, 9 and 11. To operate the mobile robot, we configure the mobile robot states (Forward, Stop, Backward, Left turn and Right turn) using services such as Pin-pair and Pin-compare. Table 4 shows how to change data for the different pins to control the motor driver using Pin-compare, which compares the received UART data to the command (between 0 and 4). The application design workflow is described in Figure 8.

### 5.3. Portability

**Figure 10:**
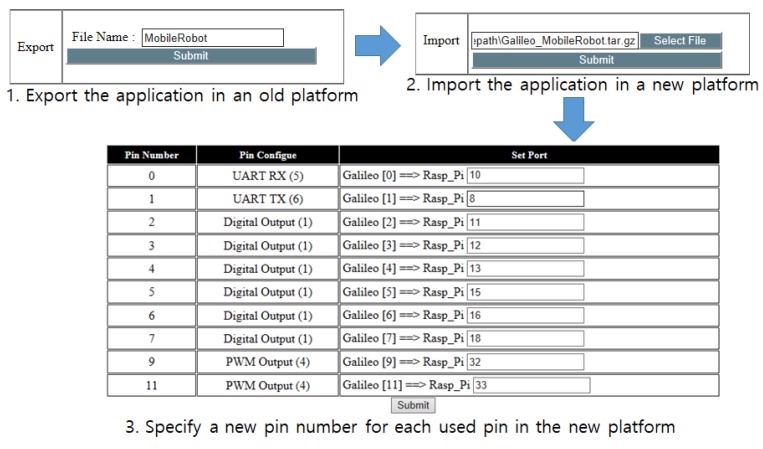
Porting the mobile robot control application for Galileo Gen 2 to Raspberry Pi.

We evaluated *SoEasy* from the aspect of portability. We show the whole procedure to port the mobile robot control application developed for Galileo Gen 2 in Section 5.2 to a Raspberry Pi platform in Figure 10. After exporting the application from an old Galileo Gen 2, we imported the exported application in a new Raspberry Pi platform. The last step is to update the pin numbers used for the new platform, as seen in the third step in Figure 10. The whole procedure consists of just a few simple mouse clicks and typing in the export file name; then, the *SoEasy* framework executes the other difficult procedures for the user.

## 6. Conclusions

Widespread open-source hardware platforms facilitate the development of diverse and novel IoT applications. However, programming various hardware platforms is still difficult for inexperienced users, since the ways to configure I/O peripherals, or the libraries that do so, are different, depending on the hardware platform. Even though there are some approaches to help users develop IoT applications easily, we determined the requirements (i.e., easiness, extensibility and portability) for IoT application programming environments from a literature review. To address the requirements, we proposed and designed the *SoEasy* framework, implemented it and evaluated its efficacy. Implementation results show the feasibility of *SoEasy* as an easy, extensible and portable programming environment. Application development in a conventional programming environment requires editing of source code and compilation to make an executable file, whereas *SoEasy* provides an easy and convenient programming environment with just a few clicks of a mouse and based on a widespread Web interface. Another useful feature of *SoEasy* is the monitoring function. Without any mobile applications, *SoEasy* provides a monitoring environment based on Web technology.

## Figures and Tables

**Table 1:** Database field.

DB Table	Table Fields
Pin-digital	Pin number, Digital pin data
Pin-analog	Pin number, Analog pin data
Pin-compare	Pin number, Comparison operators (>=, <=, <, >, ==), Comparison value, Target pin, Pin data
Pin-mode	Pin number, Pin mode (Digital, Analog, PWM, UART)
Pin-pair	Paired pins
Pin-name	Pin number, Pin name
Dutycycle-step	Pin number, Dutycycle-step
Group-control	Group-control name, Pin number, Pin data
Tik-Tok	Pin number, Timer count index, Pin data, Toggle index, Default timer index, Repeat index
Module-index	Module name, Data for portability

**Table 2:** Comparison *SoEasy* with the existing Web-based and mobile application-based IoT development frameworks. (O: Yes or Satisfactory, Δ: Average or Some, X: Deficient).

Requirements	*SoEasy*	Webduino	WebIOPi	Temboo	Blynk
Multi platforms	Raspberry Pi 2/3 Intel Galileo 1/2	Arduino with WiFi	Raspberry Pi 2/3	Arduino with WiFi Samsung ARTIC TI CC3220	Arduino Raspberry Pi Particle Photon SparkFun Blynk
ease	O	O	O/Δ	Δ	O
extensibility	O	X	X	X	X
portability	O	O/Δ	X	O	O

**Table 3:** Mobile robot control application pin configurations.

Pin Number	*SoEasy* Mode	Usage
0	UART Rx (5)	Bluetooth Tx
1	UART Tx (6)	Bluetooth Rx
2	Digital (1)	Motor Driver STBY
3	Digital (1)	Motor 1 A IN
4	Digital (1)	Motor 2 A IN
6	Digital (1)	Motor 1 B IN
7	Digital (1)	Motor 2 B IN
9	PWM (4)	Motor 1 PWM
11	PWM (4)	Motor 2 PWM

**Table 4:** Mobile robot control commands [29].

UART RX	Operation	Motor 1 A IN	Motor 2 A IN	Motor 1 B IN	Motor 2 B IN	Motor 1 PWM	Motor 2 PWM
0	Forward	High	High	Low	Low	5,000,000	5,000,000
1	Stop	High	High	High	High	0	0
2	Backward	Low	Low	High	High	5,000,000	5,000,000
3	Left Turn	High	High	Low	High	5,000,000	0
4	Right Turn	High	High	High	Low	0	5,000,000

## Abbreviations

The following abbreviations are used in this manuscript:

API	Application programming interface
GPIO	General purpose input and output
IoT	Internet of Things
UART	Universal asynchronous receiver and transmitter
PWM	Pulse width modulation
